# A Comparative *In Vitro* Study of the Effects of Separate and Combined Products of *Citrus e fructibus* and *Cydonia e fructibus* on Immunological Parameters of Seasonal Allergic Rhinitis

**DOI:** 10.1155/2012/109829

**Published:** 2012-01-19

**Authors:** E. W. Baars, M. C. Jong, I. Boers, A. F. M. Nierop, H. F. J. Savelkoul

**Affiliations:** ^1^Department of Healthcare and Nutrition, Louis Bolk Institute, 3972 LA Driebergen, The Netherlands; ^2^Muvara bv, 2353 PH Leiderdorp, The Netherlands; ^3^Department of Cell Biology and Immunology, Wageningen University, 6700 AH Wageningen, The Netherlands

## Abstract

This paper examined the effects of the combined product, *Citrus e fructibus/Cydonia e fructibus* (Citrus/Cydonia; Citrus and Cydonia: each 0.01 g/mL), and separate products of Citrus (0.01 g/mL) and Cydonia (0.01 g/mL) on the immunological pathways involved in seasonal allergic rhinitis (SAR). Peripheral blood mononuclear cells (PBMCs) from five healthy and five grass pollen-allergic donors were isolated and analyzed *in vitro* after polyclonal and allergen-specific stimulation of T cells in the presence of the three extracts. The analyses demonstrated acceptable cell survival with no signs of toxicity. Citrus mainly had a selective effect on reducing allergen-specific chronic inflammatory (TNF-*α*; Citrus compared to Cydonia and Citrus/Cydonia: −87.4 (*P* < 0.001) and −68.0 (*P* < 0.05), resp.) and Th2 pathway activity (IL-5; Citrus compared to Cydonia: −217.8 (*P* < 0.01); while, both Cydonia and Citrus/Cydonia mainly affected the induction of the allergen-specific Th1 pathway (IFN-*γ*; Cydonia and Citrus/Cydonia compared to Citrus: 3.8 (*P* < 0.01) and 3.0 (*P* < 0.01), resp.). Citrus and Cydonia demonstrated different working mechanisms in the treatment of SAR and the combination product did not demonstrate larger effects than the separate preparations. Further effectiveness and efficacy studies comparing the effects of the products on SAR *in vivo* are indicated.

## 1. Introduction

Citrus/Cydonia is an anthroposophic medicine, which contains extracts of lemon (*Citrus limon* (L.) Burm. f.) and quince (*Cydonia oblonga *Mill.) [1]. For over eighty years, the combination of preparations from Citrus and Cydonia has been prescribed as a subcutaneous injection or nasal spray for patients who suffer from seasonal allergic rhinitis (SAR). Both *in vitro* [2, 3] and clinical studies [4–6] have demonstrated that Citrus/Cydonia might be effective in treating SAR. Previous immunological analyses demonstrated it has an effect on the induction of regulatory (IL-10 producing) T-cell subsets and on the suppression of the Th2 pathway cytokines, IL-4 and IL-5 [2], *in vitro*. Another *in vitro* study demonstrated that Citrus/Cydonia significantly reduced the histamine production and the inflammatory mediator release from mast cells in a dose-dependent manner [3]. Although positive immunomodulating activity has been reported for the combination product, Citrus/Cydonia, the extent that each of the two active substances, Citrus and Cydonia, contribute to the observed effects is not known.

In this *in vitro* study, we compared the effects of the combination product, Citrus/Cydonia, and the separate products, Citrus and Cydonia, on SAR-related immunological components. The primary hypothesis of the present study, based on traditional use in clinical practice, was that the combination preparation Citrus/Cydonia would demonstrate larger SAR-related treatment effects *in vitro* than the single preparations of Citrus and Cydonia. The secondary hypothesis was that treatment with Citrus, Cydonia, and Citrus/Cydonia would demonstrate larger treatment effects (as expected in SAR patients) *in vitro, *in the healthy group compared to the SAR group. This hypothesis was based on the assumption that the three products support the SAR-related self-healing capacity of the organism and that this capacity is already stronger in healthy persons than in SAR patients before treatment.

## 2. Materials and Methods

This study was conducted according to the study protocol previously accepted by the medical ethical committee. No violations or deviations to the study protocol were noticed.

### 2.1. Investigational Products

The three preparations were obtained from WALA Heilmittel GmbH (Bad Boll/Eckwälden, Germany). Citrus/Cydonia (*Citrus e fructibus/Cydonia e fructibus*, batch number 008 704C) is a solution for subcutaneous injection. One ampoule of 1 mL contains: 0.1 g Citrus medica ssp. limonum e fructibus ferm 33c Dil. D1 HAB, prescription 33c and 0.1 g *Cydonia oblonga *e fructibus ferm 33b Dil. D1 HAB, prescription 33b. Excipients were sodium chloride, sodium hydrogen carbonate and water for injection. Citrus (Citrus e fructibus, batch no 008 702C) is a solution for subcutaneous injection. One ampoule of 1 mL contains: 1 g of Citrus medica ssp. limonum e fructibus ferm 33c Dil. D2 HAB, prescription 33c in a sodium chloride, sodium hydrogen carbonate solution for injection. Cydonia (Cydonia e fructibus, batch number 008 703C) is a solution for subcutaneous injection. One ampoule of 1 mL contains 1 g of *Cydonia oblonga *e fructibus ferm 33b Dil. D2 HAB, prescription 33b in a sodium chloride, sodium hydrogen carbonate solution for injection.

### 2.2. Blood Donors and Preparation of Blood Samples

Blood was collected from two groups of participants: SAR patients and healthy persons. The experiments performed were approved by the local medical ethical committee, and informed consent was obtained before sample collection.

Eligible participants from the SAR group were all adults of both sexes, aged 18 to 40, who gave written informed consent; suffered from SAR for at least two years; had a RAST score ≥2 for both grass pollen and birch pollen; suffered from the following nasal symptoms: sneezing, itchy nose, nasal obstruction, and watery nasal discharge; with a severity score ≥2 for at least three of the four symptoms (ranging from 0 = not present to 3 = severe) and the necessity to use antihistamines and/or corticosteroids for the treatment of symptoms for at least the last two years. Exclusion criteria were chronic inflammatory autoimmune diseases such as type I diabetes mellitus, rheumatoid arthritis, multiple sclerosis, psoriasis, or Crohn's disease; being allergic (hypersensitive) to one of the constituents of Citrus e fructibus/Cydonia e fructibus; asthma; use of other preparations containing Citrus and/or Cydonia extracts within the last two weeks prior to enrolment into the study; use of cromoglycates in the last month before study onset; concomitant pharmacological treatment indicated for seasonal allergic rhinitis such as antihistamines, corticosteroids, or other preparations in the last two weeks before study onset; antiallergy immunotherapy in the previous two years; participation in a further clinical trial at the same time or within the previous 4 weeks prior to enrolment into this study; pregnancy or lactation; severe internal or systemic disease (e.g., cardiac, hepatic, renal diseases); a known history of drug, alcohol, and/or medication dependence or addiction.

Eligible participants from the healthy group were men and women; aged 18 to 40 who gave written informed consent; had an RAST score for SAR-related pollen = 0; did not have the following nasal symptoms during the pollen season: sneezing, itching nose, nasal obstruction, and watery nasal discharge; no history of SAR symptoms for at least two years. Exclusion criteria: chronic inflammatory autoimmune disease such as type I diabetes mellitus, rheumatoid arthritis, multiple sclerosis, psoriasis, or Crohn's disease; allergic (hypersensitive) to one of the constituents of Citrus e fructibus/Cydonia e fructibus; asthma; participation in a further clinical trial at the same time or within the previous 4 weeks prior to enrolment into this study; pregnancy or lactation; severe internal or systemic disease (e.g., cardiac, hepatic, renal diseases); drug, alcohol, and/or medication dependence or addiction.

All eligible participants were recruited from a single center, the Louis Bolk Institute (Driebergen, NL). The first participant was included on June 24, 2010, and the last participant completed the study on August 3, 2010. Blood samples were taken on July 22, 2010 (2 participants), July 30, 2010 (5 participants), and August 3, 2010 (3 participants). From each person in the SAR group and healthy group, 3 × 8 mL of blood was collected in sodium heparinate-coated vacutainers (BD Biosciences, San Diego, CA, USA). The blood was subsequently diluted 1 : 1 with Iscove's Modified Dulbecco's Media (IMDM) containing GlutaMAX (IMDM; Gibco-BRL, Paisley, Scotland) before density gradient centrifugation using Ficoll-Paque PLUS (Amersham Biosciences, Uppsala, Sweden). The PBMC layer was washed twice with IMDM and the cell viability and cell concentration were determined by Trypan blue exclusion.

### 2.3. Culture Conditions and Stimulations

PBMCs were cultured in Yssel's medium at 37°C in a humidified atmosphere with 5% CO_2_ at a density of 1 × 10^6^ viable cells/mL. Immunological phenotyping of freshly isolated PBMC was performed on an FACS Canto II (BD Pharmingen, San Diego, USA), using monoclonal antibodies and the procedure from BD Pharmingen (San Diego, USA). Cells were plated out in 48 well plates at a concentration of 1 × 10^6^ cells/mL and cultured at 37°C. After five hours, in which the cells adapted to the culture conditions, various stimuli or a matching volume of medium were added. Cultures were stimulated polyclonally with 150 ng/mL anti-CD3 plus 100 ng/mL anti-CD28 monoclonal antibodies (BD Pharmingen, San Diego, CA, USA) or cultured in medium only [7]. In addition, we performed allergen-specific stimulation of 10^6^ cells/mL in 1 mL cultures with applied pollen extract (Phl p 1 from Timothy grass, Phleum pretense; Biomay Vienna, Austria; 10 *μ*g/mL in medium). Supernatants of the stimulated cultures were harvested after 1, 4, and 7 days of culture and stored at −80°C for later cytokine analysis.

### 2.4. Stimulation with Citrus/Cydonia, the Single Products of Citrus and Cydonia, Relative to Medium Control

Three experimental conditions and one control condition were evaluated. Experimental conditions: (a) Citrus, 0.01 g/mL; (b) Cydonia, 0.01 g/mL; (c) Citrus/Cydonia, 0.02 g/mL (Citrus and Cydonia: each 0.01 g/mL). The (negative) control condition and dilution medium was Yssel's medium. Samples of culture supernatants were taken after 24 hrs to elucidate monocyte reactivity, after four days to evaluate the effects of medium and polyclonal stimulation, and after seven days to evaluate the effects of the three extracts, Citrus, Cydonia, and Citrus/Cydonia, relative to a medium control for grass pollen-specific stimulation. Cytokine concentrations were measured in culture supernatants using labeled antibody preparations and flow cytometry measurements.

### 2.5. Cell Viability

Early apoptosis and late apoptosis/necrosis was assessed on freshly prepared and 7-day cultured cells using double staining with APC-Annexin V and propidium iodide (PI) [8]. On day 0 half a million cells from the isolated PBMCs of the individuals were washed and subsequently incubated with 2 *μ*L Annexin V-APC (BD Biosciences, San Diego, CA, USA) in 200 *μ*L Annexin V buffer according to the manufacturer's protocol. After a 15 min incubation period, the cells were spun down (400 g for 10 min) and resuspended in 200 *μ*L Annexin V buffer and 2 *μ*L PI (1 mg/mL; Sigma, St. Louis, Mo, USA). The cells were then analyzed on a flow cytometer (FACS array, BD Biosciences, San Diego, CA, USA) as detailed in Sections 2.6 and 2.7.

### 2.6. Immunological Phenotype

On day 0 the immunological phenotype of PBMC subsets was determined by staining the surface antigens with the following two monoclonal antibody (**α**) mixtures: (1) **α**-hCD3 (PE-Cy7), **α**-hCD4 (PE), **α**-hCD8 (APC), and **α**-hCD25 (APC-Cy7); (2) **α**-hCD3 (PE-Cy7), **α**-hCD14 (APC), **α**-hCD16 (PE), **α**-hCD19 (APC-Cy7), and **α**-hCD56 (PE). All antibodies were purchased at BD Biosciences (San Diego, CA, USA). For each well, 5 × 10^5^ cells were spun down in a 96 well U-bottom plate. The cells were incubated with staining buffer (1% FCS and 0.1 M NaN_3_ in PBS) containing the surface markers or the matching isotype controls for 30 min on ice in the dark. The cells were washed once with PBS and resuspended in PBS for flow cytometry. The four-color flow cytometric acquisition was performed on an FACS array, using the BD FACS-array software. An electronic gate was set to exclude debris and at least 10,000 events/samples were acquired. The percentage of positive cells was corrected for the isotype control.

### 2.7. Proliferation Capacity

On days 4 and 7 the proliferation capacity of the PBMCs was studied by intracellular expression of the nuclear Ki-67 antigen (Ki-67; BD Pharmingen, San Diego, CA, USA). The Ki-67 antigen is absent in the nuclei of resting cells, but present in all other phases of the cell division cycle as well as in the mitosis phase [8, 9]. In each well, 5 × 10^5^ PBMCs were incubated with 100 *μ*L Cytofix/Cytoperm (BD Pharmingen, San Diego, CA, USA) for 15 to 20 min on ice to fix and permeabilize the cells. Cells were washed twice with perm/wash buffer (BD Pharmingen, San Diego, CA, USA) and incubated with anti-Ki-67 PE antibody or the matched isotype control, diluted in perm/wash buffer, for 30 min on ice in the dark. Hereafter, the cells were washed with perm/wash buffer, resuspended in PBS, and measured on the flow cytometer. Values are expressed as cells positive for the Ki-67 mAb, corrected for the isotype control.

### 2.8. Cytokines

On days 1, 4, and 7 PBMC culture supernatants were analyzed for their IL-1*β*, IL-12, IFN-*γ*, TNF-*α*, IL-5, IL-10, and IL-13 contents. The cytokine production was measured with Cytometric Bead Assay Flex Sets (BD Pharmingen, San Diego, CA, USA). All buffers used in this protocol were obtained from the BD CBA Soluble Protein Master Buffer Kit (BD Pharmingen, San Diego, CA, USA). Supernatants were collected, stored at −20°C, and tested within 2 weeks. The procedure was performed according to the manufacturer's protocol. The samples were measured on the FACS array, using the FCAP software. The sensitivity limits for quantitative determinations, according to the manufacturer, were 1.1 pg/mL for IL-1*β*, 0.3 pg/mL for IFN-*γ*, 0.5 pg/mL for IL-5, 2.3 pg/mL for IL-10, 2.2 pg/mL for IL-12, 0.6 pg/mL for IL-13, and 0.7 pg/mL for TNF-*α*.

### 2.9. Definition of Positive Effects

**Figure 1:**
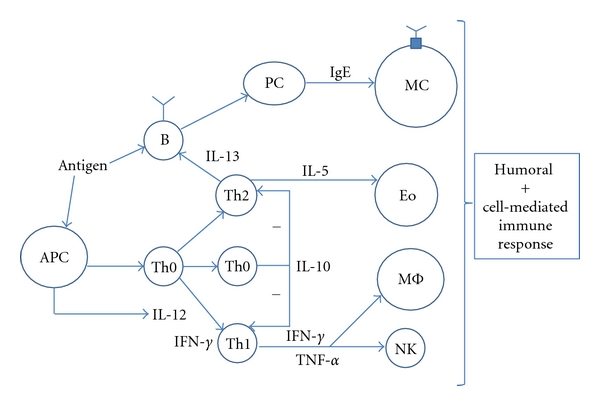
Seasonal allergic rhinitis related immunological subsystems, pathways, and cytokines.

A nonallergic, healthy state is hypothesized to represent a balanced state within the immune system with a relatively high level of IL-10 (demonstrating sufficient immunoregulation) and a balance between the Th1 pathway (e.g., IFN-*γ*) and the Th2 pathway (e.g., IL-5 and IL-13). SAR is associated with relatively low levels of IL-10, a chronic inflammatory activity (e.g., TNF-*α*), an overproduction of Th2 pathway cytokines, and an imbalance between the Th1 and Th2 pathways [9, 10]. The following changes in cytokine production levels were therefore regarded as positive immunological SAR treatment effects [9–14] (Figure 1).

1. The induction of (regulatory) T cells (Treg): increase of (grass pollen stimulated minus medium stimulated) IL-10 on day 7, often accompanied by monocyte-derived IL-10 on day 1.
2. The induction of Th1 activity: increase of (grass pollen stimulated minus medium stimulated) IFN-*γ* on day 7, often accompanied by monocyte-derived IFN-*γ* on day 1.
3. The reduction of Th2 activity: reduction of (grass pollen stimulated minus medium stimulated) IL-5 and IL-13 on day 7, often accompanied by monocyte-derived IL-1*β* on day 1, which is essential for the outgrowth of Th2 cells.
4. The reduction of chronic inflammatory activity: reduction of (grass pollen stimulated minus medium stimulated) monocyte-derived TNF-*α* on day 1.
5. The restoration of the Th1/Th2 balance: an increase in (grass pollen stimulated minus medium stimulated) IFN-*γ*/IL-5 and IFN-*γ*/IL-13 ratios on day 7.
6. The restoration of the Treg/Th2 balance: an increase in the (grass pollen stimulated minus medium stimulated) IL-10/TNF-*α* ratio on day 1; the increase of (grass pollen stimulated minus medium stimulated) IL-10/IL-5 and IL-10/IL-13 ratios on day 7.

### 2.10. Statistics

Pearson chi-square tests were performed on all relevant donor characteristics. For the PBMC subsets, we calculated all immunological outcome parameters (day 1: IL-1*β*, IL-10, IL-12, TNF-*α*, and IFN-*γ*; day 7: IL-5, IL-13, IL-10, and IFN-*γ*), the relevant ratios (day 1: IL10/TNF-*α* and IFN-*γ*/TNF-*α*; day 7: IL10/IL-5, IL-10/IL-13, IFN-*γ*/IL-5, and IFN-*γ*/IL-13), the mean scores, and 95% confidence intervals of cytokine production levels after grass pollen stimulation by subtracting the medium stimulation values. Subsequently, ANOVA (with Tamhane correction in case of unequal distribution) and unpaired *t*-tests were used to determine statistically significant differences between (1) the means of the three SAR groups that were stimulated in the presence of the three experimental extracts, (2) the means of the three healthy groups that were stimulated in the presence of the three experimental extracts, and (3) the means of the SAR groups and the healthy groups that were stimulated in the presence of the three experimental extracts. GLM Repeated Measures tests were performed to determine statistically significant differences between the means of the three total groups (total group = SAR group and healthy group) that were stimulated in the presence of the three experimental extracts. All statistical analyses were performed with SPSS 18.0 (SPSS Inc., Chicago, USA).

### 2.11. Blinding

All researchers were blinded to the identity of the products, which were numbered A, B, and C by the manufacturer. Unblinding took place after all statistics had been performed.

## 3. Results

### 3.1. Blood Donors

The SAR group and the healthy group demonstrated no statistically significant differences with regard to sex (both groups: 3 women and 2 men); ethnicity (both groups: all Caucasian); age (means and standard deviations): 35.2 (10.5) and 43.4 (10.9) years, respectively; height (means and standard deviations): 1.79 (0.1) and 1.77 (0.1) meters, respectively; weight (means and standard deviations): 75 (12.6) and 74 (9.2) kg, respectively; systolic blood pressure (means and standard deviations): 113 (13.2) and 117 (12.0) Hg, respectively; diastolic blood pressure (means and standard deviations): 71 (7.5) and 78 (5.7) Hg, respectively; and heart rate (means and standard deviations): 65 (3.8) and 65 (8.7), respectively. RAST scores (means, standard deviations and ranges) for grass pollen and birch pollen respectively were 3.6 (1.5, range: 2–6) and 3.6 (0.9, range: 3–5) for the SAR group, and 0 and 0 for the healthy group (Table 1).

 The mean percentages of the PBMC subsets for the SAR group were 58.8% CD3+ T cells (with 40.4% CD4+ Th cells and 18.4% CD8+ Tc cells), 6.2% CD19+ B cells, 16% CD14+ monocytes, and 9.6% CD16/CD56+ NK cells. The mean percentages of the PBMC subsets for the healthy group were 60.0% CD3+ T cells (with 39.4% CD4+ Th cells and 20.6% CD8+ Tc cells), 7.0% CD19+ B cells, 15.8% CD14+ monocytes, and 8.8% CD16/CD56+ NK cells. There were no statistically significant differences in the mean scores of the subsets between the SAR group and the healthy group.

### 3.2. Effects on Viability

**Figure 2:**
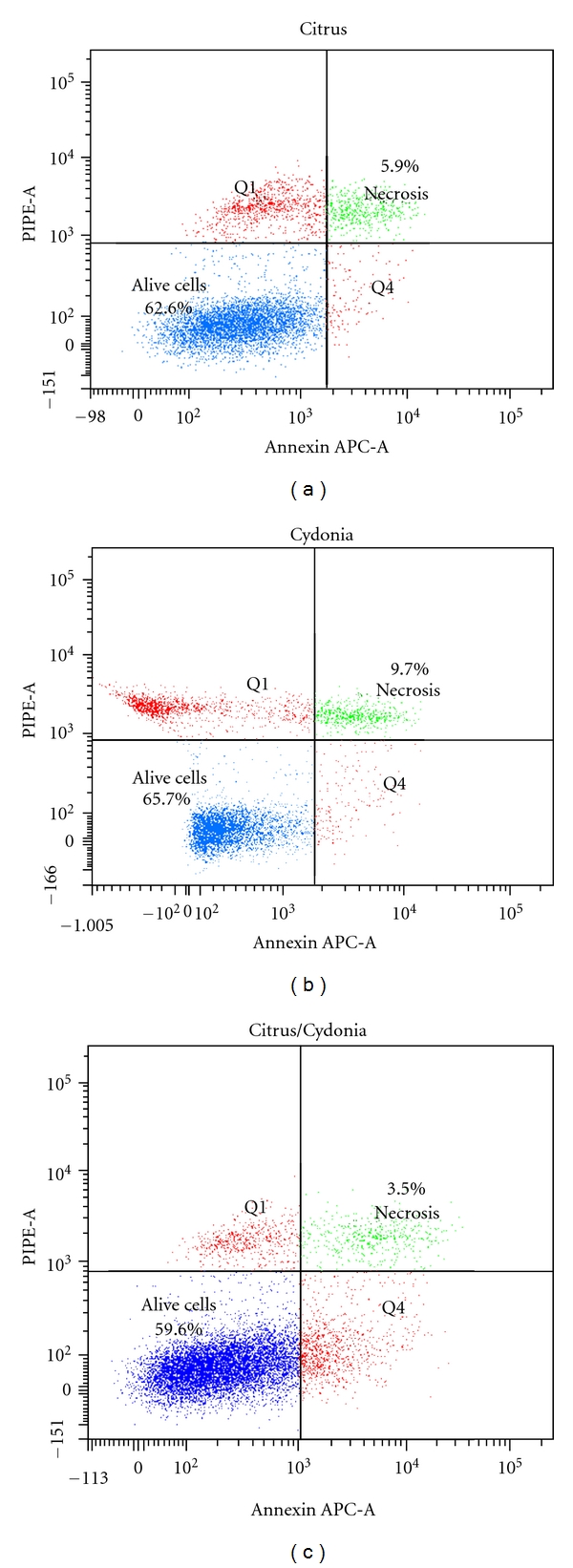
Representative example of a flow cytometric analysis profile of live, necrotic, and apoptotic cells after *in vitro* culture of human PBMC stimulated in the presence of either Citrus (a) or Cydonia (b) or their combination (c). Citrus (100 *μ*L/mL), Cydonia (100 *μ*L/mL), or the combination Citrus/Cydonia (100 *μ*L/mL) were added to 0.5 × 10^6^ PBMC in 1 mL cultures which were stimulated with grass pollen extract (10 mg/mL) for 7 days. Staining was performed with Annexin V-APC and PI and measured on a BD flow cytometer.

Citrus (Figure 2(a)), Cydonia (Figure 2(b)), and Citrus/Cydonia (Figure 2(c)) had no effect on *in vitro* cultured blood mononuclear cell survival and did not appear to be toxic to the PBMC subpopulations by Annexin V-PI staining.

### 3.3. Effects on PBMCs from Allergic Donors

**Figure 3:**
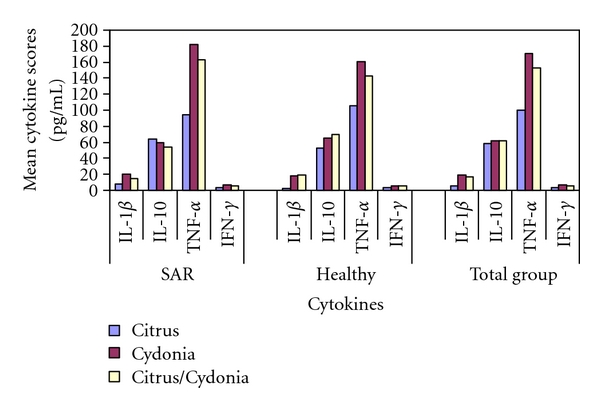
Summary of differences in mean scores after one-day PBMC cultures in the SAR group, healthy group, and total group.

**Figure 4:**
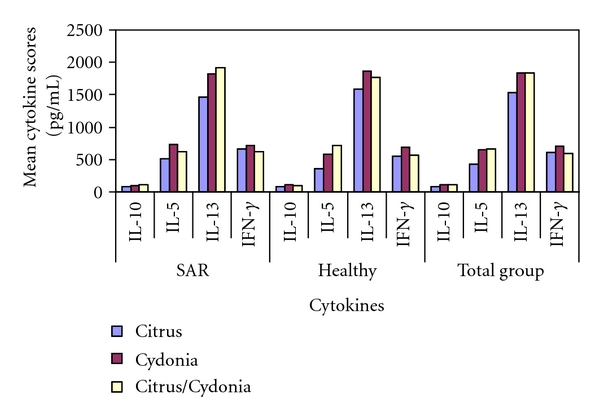
Summary of differences in mean scores after seven days PBMC cultures in the SAR group, healthy group, and total group.

The mean cytokine scores after one-day culture and seven-day culture are presented in Tables 2 and 3, respectively, and Figures 3 and 4, respectively. Citrus demonstrated a larger reduction of chronic inflammatory activity than Cydonia and Citrus/Cydonia (TNF-*α* (day 1): −87.4 (95% CI: −120.3 to −54.5), *P* < 0.001) and −68.0 (95% CI: −100.9 to −35.1), *P* < 0.05, resp.) and had a larger effect on the restoration of the allergen-specific Treg/Th2 balance (IL-10/TNF-*α* (day 1): 0.34 (95% CI: 0.20 to 0.49), *P* < 0.001 and 0.32 (0.17 to 0.47), *P* < 0.01, resp.). Citrus was also more powerful than Cydonia in the reduction of Th2 pathway activity in both the innate reaction (IL-1*β* (day 1): −12.4 (95% CI: −22.3 to −2.5), *P* < 0.05) and the outgrowth of the allergen-specific specialized T-cell subsets (IL-5 (day 7): −217.8 (95% CI: −361.9 to −73.7), *P* < 0.01). Citrus also demonstrated larger effects than Citrus/Cydonia on the reduction of the Th2 pathway activity of the allergen-specific specialized T-cell subsets (IL-13 (day 7): −447.2 (95% CI: −855.9 to 38.5), *P* < 0.05). Both Cydonia and Citrus/Cydonia demonstrated larger effects than Citrus on the innate induction of Th1 pathway activity (IFN-*γ* (day 1): 3.8 (95% CI: 1.8 to 5.8), *P* < 0.01 and 3.0 (95% CI: 1.0 to 5.0), *P* < 0.01, respectively. 

### 3.4. Effects on the PBMCs from Healthy Donors

Similar to the results in the allergic donor group (Table 2), Citrus demonstrated a larger effect in the healthy donor group than Cydonia and Citrus/Cydonia on the reduction of chronic inflammatory activity (TNF-*α* (day 1): −54.6 (95% CI: −84.2 to −25.0), *P* < 0.01 and −37.6 (95% CI: −67.2 to −8.0), *P* < 0.05, resp.) but not on the restoration of the Treg/Th2 balance (IL-10/TNF-*α*—day 1). As in the allergic donor group, Citrus was also more powerful than Cydonia in the healthy donor group in reducing Th2 pathway activity in both the innate reaction (IL-1*β* (day 1): −15.2 (95% CI: −23.7 to −6.7), *P* < 0.01) and the outgrowth of the allergen-specific specialized T-cell subsets (IL-5 (day 7): −217.8 (95% CI: −371.4 to −64.2), *P* < 0.01). Unlike the results in the allergic donor group, Citrus also demonstrated larger effects than Citrus/Cydonia on the reduction of Th2 pathway activity in both the innate reaction (IL-1*β* (day 1): −16.4 (95% CI: −24.9 to −7.9), *P* < 0.01) and the outgrowth of the specialized T-cell subsets (IL-5 (day 7): −353.4 (95% CI: −507.0 to −199.8), *P* < 0.001). Finally, Citrus was more powerful than Citrus/Cydonia in restoring the Th1/Th2 balance (IFN-*γ*/IL-5 (day 7): 0.75 (95% CI: 0.38 to 1.12), *P* < 0.01).

Both Cydonia and Citrus/Cydonia also demonstrated larger effects than Citrus on the innate induction of the Th1 pathway (IFN-*γ* (day 1): 3.0 (95% CI: 1.1 to 4.9), *P* < 0.01 and 2.4 (95% CI: 0.5 to 4.3), *P* < 0.05, resp.). In addition, Cydonia was more powerful than Citrus/Cydonia on the allergen-specific T-cell subset related induction of the Th1 pathway (IFN-*γ* (day 7): 120.6 (95% CI: 10.8 to 230.4), *P* < 0.05) and the restoration of the Th1/Th2 balance (IFN-*γ*/IL-5 (day 7): 0.38 (95% CI: −0.02 to 0.78), *P* < 0.05) (Table 3). 

### 3.5. Effects on the Total Group

As in the allergic donor group and the healthy donor group, Citrus demonstrated a larger reduction of chronic inflammatory activity than Cydonia and Citrus/Cydonia (TNF-*α* (day 1): −71.0 (95% CI: −92.5 to −49.5), *P* < 0.001 and −52.8 (95% CI: −74.3 to −31.3), *P* < 0.001, resp.). As in the SAR group, Citrus demonstrated larger effects than Cydonia and Citrus/Cydonia in the restoration of the Treg/Th2 balance (IL-10/TNF-*α* (day 1): 0.23 (95% CI: 0.11 to 0.35), *P* < 0.01 and 0.17 (95% CI: 0.05 to 0.29), *P* < 0.01, resp.). As in the allergic donor and healthy donor groups, Citrus was also more powerful than Cydonia in the reduction of Th2 pathway activity in both the innate reaction (IL-1*β* (day 1): −13.8 (95% CI: −20.0 to −7.6), *P* < 0.001) and the outgrowth of the allergen-specific T-cell subsets (IL-5 (day 7): −217.8 (95% CI: −331.8 to −103.8), *P* < 0.01). As in the healthy donor group, Citrus was also more powerful than Citrus/Cydonia in the reduction of Th2 pathway activity in both the innate reaction (IL-1*β* (day 1): −11.4 (95% CI: −17.6 to −5.2), *P* < 0.01) and the outgrowth of the allergen-specific T-cell subsets (IL-5 (day 7): −231.3 (95% CI: −345.3 to −117.3), *P* < 0.001).

Both Cydonia and Citrus/Cydonia demonstrated larger effects than Citrus on the innate induction of the Th1 pathway activity (IFN-*γ* (day 1): 3.4 (95% CI: 2.1 to 4.7), *P* < 0.001 and 2.7 (95% CI: 1.4 to 4.0), *P* < 0.001, resp.), which was also found in the allergic donor and healthy donor groups. In addition, Cydonia was more powerful than both Citrus (unlike the results of the other groups) and Citrus/Cydonia (like the results in the healthy donor group) on the allergen-specific T-cell subset related induction of Th1 pathway activity (IFN-*γ* (day 7): 95.6 (95% CI: 3.2 to 188.0), *P* < 0.05) and 111.6 (95% CI: 19.2 to 204.0), *P* < 0.05, resp.). In addition, as in the healthy donor group, both Citrus and Cydonia were more powerful than Citrus/Cydonia in the restoration of Th1/Th2 balance (IFN-*γ*/IL-5 (day 7): 0.37 (95% CI: 0.06 to 0.68), *P* < 0.01 and 0.56 (95% CI: 0.26 to 0.87), *P* < 0.001, resp.) Finally, unlike the results in the allergic donor group and the healthy donor group, Citrus/Cydonia demonstrated a larger effect on the allergen-specific T-cell subset related induction of Treg activity (IL-10 (day 7): 26.4 (95% CI: 1.1 to 51.7), *P* < 0.05) (Table 4).

### 3.6. Comparison of the Effects on PBMCs from Allergic and Healthy Donors

Statistically significant differences between the allergic donor group and the healthy donor group were demonstrated on day 1 with regard to: TNF-*α* in the Cydonia group (182.2 versus 160.2, resp., *P* < 0.05) and IL-10 (day 1) in the Citrus/Cydonia group (54.0 versus 69.6, resp., *P* < 0.05); on day 7 with regard to IL-5 in the Citrus group (509.9 versus 358.0, resp., *P* < 0.05).

### 3.7. Comparison of the Results in the Different Groups

In all three groups (allergic donor, healthy donor, and total group) Citrus consistently demonstrated a selective effect on the reduction of chronic inflammatory activity compared to the other two extracts and on the reduction of allergen-specific Th2 pathway activity (Table 4: IL-1*β*, TNF-*α*, and IL-5). Cydonia and Citrus/Cydonia consistently demonstrated a selective effect on the induction of the innate Th1 pathway activity compared to Citrus (Table 4: IFN-*γ*—day 1) in all three groups.

 In the allergic donor group and the total group, Citrus demonstrated a larger restoration of Treg/Th2 balance compared to the other two extracts (Table 4: IL-10/TNF-*α*). In the healthy donor group and the total group, Citrus induced a reduction in the activity of an allergen-specific, specialized T-cell subset related Th2 pathway compared to Citrus/Cydonia (Table 4: IL-5). Citrus also demonstrated a restoration of the Th1/Th2 balance compared to Citrus/Cydonia (Table 4: IFN-*γ*/IL-5) and Cydonia demonstrated a larger effect on the induction of the allergen-specific, specialized T-cell subset related Th1 pathway compared to Citrus/Cydonia (Table 4: IFN-*γ*-day 7). All analyses with regard to cell viability demonstrated acceptable cell survival, with no signs of toxicity, providing evidence for the safety of the three extracts (data not shown).

## 4. Discussion

In this *in vitro* study we examined the immunological effects of the combined product, *Citrus e fructibus/Cydonia e fructibu*s (Citrus/Cydonia), and separate products Citrus and Cydonia on PBMCs from a group of five healthy and five grass pollen-allergic donors to study possible differences in the working mechanisms and magnitude of the effects. Previous *in vitro* studies and clinical studies already repeatedly demonstrated positive treatment effects of Citrus/Cydonia on immunological and clinical SAR-related elements [2–6]. The primary hypothesis of the present study was that the combination product Citrus/Cydonia would demonstrate larger SAR-related immunological treatment effects *in vitro* than each of the single products. The secondary hypothesis was that the healthy group would demonstrate larger SAR-related immunological treatment effects *in vitro *than the SAR group.

 In grass pollen allergies, a state of chronic inflammation in the upper airways is reminiscent of allergen-induced activity of the innate immune system, which can be analyzed by the presence of TNF-*α* and IL-1*β* on day 1 in allergen-induced *in vitro* PBMC cultures. The allergen-induced outgrowth of various T-cell subsets *in vitro* is widely considered to be reminiscent of the presence of selected cytokines after seven days of culture, including IFN-*γ* (a signature Th1 cytokine), IL-5 (a signature Th2 cytokine), and IL-10 (a signature Treg cytokine) [9, 10, 13, 14]. Based on our results, we conclude that Citrus and Cydonia appear to have different working mechanisms. Citrus has mainly a selective effect on the reduction of chronic inflammatory activity and the reduction of allergen-specific Th2 pathway activity while Cydonia has mainly a selective effect on the induction of the innate related, allergen-specific Th1 pathway activity. In theory the combination product Citrus/Cydonia would, therefore, provide an effective therapy that targets SAR from different and possibly additive or synergistic working mechanisms. However, the empirical results demonstrated that Citrus/Cydonia does not provide larger effects than the separate components, both with regard to the reduction of the allergen-specific Th2 pathway activity and the induction of the innate related, allergen-specific Th1 pathway activity *in vitro*. Thus, the first hypothesis needs to be rejected. Based on these results, further effectiveness and (placebo) controlled efficacy studies are indicated to compare the effects of the three products on SAR *in vivo.*

The differences between the SAR group and the healthy group (TNF-*α* in the Cydonia group, IL-10 (day 1) in the Citrus/Cydonia group, and IL-5 in the Citrus group) and the comparison of the results of both groups (Table 3) demonstrates overall larger and more effects in the healthy group. These results implicate effects on both the innate arm of the immune response (monocyte-derived TNF and IL-10 production) and the potential Th2 arm of the adaptive immune response (reflected by the IL-5 effect). The second hypothesis of this study could therefore be confirmed, providing empirical underpinning of the assumption that the products support the SAR-related self-healing capacity of the organism and that this capacity is already stronger in healthy persons than in SAR patients before treatment.

The positive effects of Citrus and Cydonia are in line with fundamental studies on the immunomodulating compounds of these fruits. The extracts of Citrus [15] and Cydonia [16, 17] contain several immunologically active compounds, including organic acids, polyphenols, flavonoids, and pectins. For example, flavonoids are mainly present in Citrus fruits as their glycosyl derivatives [18]. Flavonoids appear to be able to regulate acute and chronic inflammatory responses and to suppress production of tumor necrosis factor-*α* (TNF-*α*), by macrophages, microglial cells, and mast cells stimulated with lipopolysaccharide (LPS) and others via Toll-like receptors (TLRs), and TNF-*α*-mediated acute and chronic inflammatory responses [19–21]. And for example, the pectins in Cydonia extract generally contain a galacturonic acid. This immunomodulatory compound may strengthen innate-immune responses that may be beneficial in specific conditions, such as attenuation of allergic disease [22, 23].

The major limitation of this study is that it is an *in vitro* study that provides, within the classic evidence hierarchy framework, only limited evidence on the effectiveness of the examined extracts *in vivo*. However, the results are in line with previous *in vitro *studies [2, 3] and an *in vitro *study within an *in vivo* randomized controlled trial [5], demonstrating the positive SAR-related immunological effects of Citrus/Cydonia.

## 5. Conclusion

It appears that Citrus and Cydonia have different working mechanisms in the treatment of SAR *in vitro*. Citrus mainly inhibits the chronic inflammatory activity and the SAR-related Th2 pathway activity whereas Cydonia mainly promotes the SAR-related Th1 pathway activity. Theoretically, the combination of both extracts would provide an optimal treatment that would target SAR from different directions. However, in this *in vitro* study, the combination product Citrus/Cydonia did not demonstrate larger effects than Citrus and Cydonia separately.

The primary hypothesis of the present study, that the combination preparation Citrus/Cydonia as a whole would demonstrate larger SAR-related treatment effects *in vitro* than each of the single preparations, was rejected. The secondary hypothesis, that the healthy group would demonstrate larger SAR-related treatment effects *in vitro *than the SAR group, was confirmed.

Future studies could focus on the comparison of the effects of the separate extracts and the combination product *in vivo.*

## Figures and Tables

**Table 1:** Baseline characteristics seasonal allergic rhinitis (SAR) group and healthy group.

Variable		SAR group (*n* = 5)	Healthy group (*n* = 5)	*P* value
Sex: number (percentage)	Male	3 (60%)	3 (60%)	n.s.
	Female	2 (40%)	2 (40%)	
Ethnicity: number (percentage)	Caucasian	5 (100%)	5 (100%)	n.s.
	Asian	0 (0%)	0 (0%)	
Age (year) (sd)		35.2 (10.5)	43.4 (10.9)	n.s.
Height (cm) (sd)		179 (10)	177 (10)	n.s.
Weight (kg) (sd)		75 (12.6)	74 (9.2)	n.s.
Blood pressure at screening (systolic/diastolic) (mmHg) (sd)		113 (13.2)/71 (7.5)	117 (12)/78 (5.7)	n.s.
Heart rate at screening (beats per minute)		65 (3.8)	65 (8.7)	n.s.
RAST grass pollen		3.6 (1.5)	0	
RAST birch pollen		3.6 (0.9)	0	

n.s. = not significant.

**Table 2:** Differences in mean cytokine scores after one-day PBMC cultures in the SAR group, healthy group, and total group (grass pollen stimulation minus medium stimulation).

		Citrus	Cydonia	Citrus/Cydonia	Citrus versus Cydonia *P* value	Citrus versus Citrus/Cydonia *P* value	Cydonia versus Citrus/Cydonia *P* value
IL-1β*	SAR	8.2 (7.0)	20.6 (5.0)	14.6 (8.9)	*P* < 0.05	n.s.	n.s.
	Healthy	2.4 (2.3)	17.6 (6.7)	18.8 (8.0)	*P* < 0.01	*P* < 0.01	n.s.
	Total group	5.3 (5.8)	19.1 (5.8)	16.7 (8.3)	*P* < 0.001	*P* < 0.01	
IL-10	SAR	64.0 (14.4)	59.2 (10.1)	54.0 (8.2)	n.s.	n.s.	n.s.
	Healthy	53.0 (15.2)	65.2 (14.4)	69.6 (3.9)	n.s.	n.s.	n.s.
	Total group	58.5 (15.1)	62.2 (12.1)	61.8 (10.2)	n.s.	n.s.	n.s.
TNF-*α*	SAR	94.8 (9.0)	182.2 (15.2)	162.8 (37.4)	*P* < 0.001	*P* < 0.05	n.s.
	Healthy	105.6 (25.2)	160.2 (13.2)	143.2 (23.9)	*P* < 0.01	*P* < 0.05	n.s.
	Total group	100.2 (18.7)	171.2 (17.7)	153.0 (31.3)	*P* < 0.001	*P* < 0.001	
IFN-*γ*	SAR	3.0 (1.9)	6.8 (1.1)	6.0 (1.2)	*P* < 0.01	*P* < 0.01	n.s.
	Healthy	3.0 (1.6)	6.0 (1.4)	5.4 (1.1)	*P* < 0.01	*P* < 0.05	n.s.
	Total group	3.0 (1.6)	6.4 (1.3)	5.7 (1.2)	*P* < 0.001	*P* < 0.001	
IL-10/ TNF-*α*	SAR	0.7 (0.1)	0.3 (0.1)	0.4 (0.1)	*P* < 0.001	*P* < 0.01	n.s.
	Healthy	0.5 (0.2)	0.4 (0.1)	0.5 (0.1)	n.s.	n.s.	n.s.
	Total group	0.6 (0.2)	0.4 (0.1)	0.4 (0.1)	*P* < 0.01	*P* < 0.01	

* The mean score (SD) in pg/mL is presented for all cytokine measurements; n.s., not significant.

**Table 3:** Differences in mean scores after seven-day PBMC cultures in the SAR group, healthy group, and total group (grass pollen stimulation minus medium stimulation).

		Citrus	Cydonia	Citrus/Cydonia	*P* value Citrus versus Cydonia	*P* value Citrus versus Citrus/Cydonia	*P* value Cydonia versus Citrus/Cydonia
IL-10	SAR	88.4 (28.5)	101.4 (19.5)	115.2 (36.2)	n.s.	n.s.	n.s.
	Healthy	76.4 (38.2)	107.4 (16.5)	102.4 (26.1)	n.s.	n.s.	n.s.
	Total group	82.4 (32.4)	104.4 (17.3)	108.8 (30.5)	n.s.	n.s.	*P* < 0.05
IL-5	SAR	509,8 (126.6)	727.6 (95.5)	619.0 (87.7)	*P* < 0.01	n.s.	n.s.
	Healthy	358.0 (81.3)	575.8 (98.6)	711.4 (144.7)	*P* < 0.01	*P* < 0.001	n.s.
	Total group	433.9 (128.3)	651.7 (121.6)	665.2 (122.9)	*P* < 0.01	*P* < 0.001	n.s.
IL-13	SAR	1470 (390.3)	1823.0 (185.7)	1917.2 (277.7)	n.s.	*P* < 0.05	n.s.
	Healthy	1589.2 (300.2)	1864.2 (102.0)	1765.4 (186.6)	n.s.	n.s.	n.s.
	Total group	1529.6 (334.2)	1843.6 (142.9)	1841.3 (237.0)	n.s.	n.s.	n.s.
IFN-*γ*	SAR	666.8 (126.2)	724.0 (105.2)	621.4 (90.8)	n.s.	n.s.	n.s.
	Healthy	558.0 (130.1)	692.0 (58.4)	571.4 (57.1)	n.s.	n.s.	*P* < 0.05
	Total group	612.4 (133.7)	708.0 (82.0)	596.4 (76.2)	*P* < 0.05	n.s.	*P* < 0.05
IFN-*γ*/ IL-5	SAR	1.4 (0.5)	1.0 (0.2)	1.0 (0.2)	n.s.	n.s.	n.s.
	Healthy	1.6 (0.4)	1.2 (0.2)	0.8 (0.2)	n.s.	*P* < 0.01	*P* < 0.05
	Total group	1.5 (0.4)	1.1 (0.2)	0.9 (0.2)	*P* < 0.01	*P* < 0.001	n.s.
IFN-*γ*/ IL-13	SAR	0.5 (0.1)	0.4 (0.1)	0.3 (0.1)	n.s.	n.s.	n.s.
	Healthy	0.4 (0.1)	0.4 (0.1)	0.3 (<0.1)	n.s.	n.s.	n.s.
	Total group	0.4 (0.1)	0.4 (0.1)	0.3 (0.1)	n.s.	n.s.	n.s.
IL-10/ IL-5	SAR	0.2 (0.1)	0.1 (<0.1)	0.2 (0.1)	n.s.	n.s.	n.s.
	Healthy	0.2 (0.1)	0.2 (0.1)	0.2 (0.1)	n.s.	n.s.	n.s.
	Total group	0.2 (0.1)	0.2 (<0.1)	0.2 (0.1)	n.s.	n.s.	n.s.
IL-10/ IL-13	SAR	0.1 (<0.1)	0.1 (<0.1)	0.1 (<0.1)	n.s.	n.s.	n.s.
	Healthy	<0.1 (<0.1)	0.1 (<0.1)	0.1 (<0.1)	n.s.	n.s.	n.s.
	Total group	0.1 (<0.1)	0.1 (<0.1)	0.1 (<0.1)	n.s.	n.s.	n.s.

* The mean score (SD) in pg/mL is presented for all cytokine measurements; n.s., not significant.

**Table 4:** Summary of effects on days 1 and 7 in the SAR group, healthy group, and total group.

Cytokines (immunological SAR-related subset) on day 1 and day 7	SAR group			Healthy group			Total group		
	CI versus CY	CI versus CI/CY	CY versus CI/CY	CI versus CY	CI versus CI/CY	CY versus CI/CY	CI versus CY	CI versus CI/CY	CY versus CI/CY
*Day 1*									
IL-1*β* (Th2)	CI*			CI**	CI**		CI***	CI**	
TNF-*α* (Chr. Inf.)	CI***	CI*		CI**	CI*		CI***	CI***	
IFN-*γ* (Th1)	CY**	CI/CY**		CY**	CI/CY*		CY***	CI/CY***	
IL-10/TNF-*α* (Treg/Chr. Inf)	CI***	CI**					CI**	CI**	
*Day 7*									
IL-10 (Treg)								CI/CY*	
IL-5 (Th2)	CI**			CI**	CI***		CI**	CI***	
IL-13 (Th2)		CI*							
IFN-*γ* (Th1)						CY*	CY*		CY*
IFN-*γ*/IL-5 (Th1/Th2)					CI**	CY*	CI**	CI***	

*: *P* < 0.05; **: *P* < 0.01; ***: *P* < 0.001; CI = Citrus; CY = Cydonia; CI/CY = Citrus/Cydonia, Treg = regulatory T cells; Th2 = SAR-related Th2 pathway; Th1 = SAR-related Th1 pathway; Chron. Inf. = SAR-related chronic inflammatory activity. Example: CI** (CI versus CY): Citrus (in relation to Cydonia) demonstrates an SAR-related treatment effect with a statistically significant difference < 0.01.
